# Microskills Bootcamp for Ultrasound Training

**DOI:** 10.7759/cureus.106143

**Published:** 2026-03-30

**Authors:** Jesse Z Kellar, Ashley Garispe, Sameir Alhadi, Adam Macksoud, Erick Melara

**Affiliations:** 1 Emergency Department, Saint Agnes Medical Center, Fresno, USA

**Keywords:** accreditation council for graduate medical education (acgme), basic skills, microskills, research in emergency medicine, residency program, teaching bootcamp, ultrasound

## Abstract

Introduction: We evaluated the impact of targeted ultrasound instruction on learner comfort following a pre-post observational microskill bootcamp focused on foundational ultrasound image acquisition, interpretation, and ultrasound-guided procedural skills.

Methods: Pre- and postintervention Likert (1-5) comfort ratings across four domains were paired by learner and analyzed using two-sided paired t-tests with 95% confidence intervals (CIs). Wilcoxon signed-rank testing was performed as a nonparametric sensitivity analysis.

Results: Significant improvements were observed across all items (n = 14 pairs): Q1 Δ = +0.929 (0.573-1.284), p < 0.001; Q2 Δ = +0.714 (0.238-1.191), p = 0.006; Q3 Δ = +1.714 (1.187-2.242), p < 0.001; Q4 Δ = +1.800 (1.371-2.229), p < 0.001. Effect sizes were large (Cohen’s dz = 0.865-2.324).

Conclusion: Targeted ultrasound instruction delivered through a microskill-based teaching approach was associated with large gains in learner comfort.

## Introduction

Point-of-care ultrasound (POCUS) has become an increasingly important bedside tool across multiple specialties because it allows clinicians to rapidly answer focused diagnostic questions and guide procedures in real time. Effective POCUS use requires integration of image acquisition, hand-eye coordination, machine operation, and immediate clinical interpretation, making it a complex skill set for novice learners to develop [1,2]. Prior studies have shown that structured teaching, deliberate practice, and simulation-based instruction can improve learner confidence, image acquisition skills, knowledge retention, and procedural success [3-6]. These findings support the value of early, intentional training models for learners who are still developing foundational bedside skills.

In graduate medical education, the postgraduate year 1 (PGY-1) year represents a particularly important period for ultrasound instruction. During this stage, residents are developing core approaches to physical examination, procedural technique, and clinical decision-making. Early exposure to POCUS may help normalize ultrasound as part of routine patient assessment before specialty-specific responsibilities and workflow pressures intensify. This may be especially relevant for learners in transitional year (TY), internal medicine (IM), and emergency medicine (EM) programs, where baseline exposure to ultrasound can vary considerably. Although prior studies have demonstrated the benefits of ultrasound curricula in medical students and training settings [3-5], fewer studies have examined early ultrasound instruction across multiple PGY-1 training programs using a microskill-based teaching approach.

In this educational model, complex ultrasound tasks are decomposed into smaller, discrete foundational skills that are practiced stepwise in isolated stations. Rather than expecting novice learners to immediately perform complete ultrasound examinations or fully integrated ultrasound-guided procedures, this approach emphasizes progressive development of core skills such as probe handling, image acquisition, needle visualization, and image optimization. Such a strategy may be particularly well suited to early learners, for whom the psychomotor and visuospatial demands of ultrasound can otherwise be overwhelming. This approach also aligns with Ericsson’s framework of deliberate practice, in which complex skills are developed through focused, repetitive practice of specific component tasks with progressive refinement before full task integration [6]. By breaking ultrasound down into foundational subskills practiced in isolation, the microskills model provides a pedagogically grounded strategy for early skill acquisition and progressive mastery development.

To address this gap, we implemented a microskill-based POCUS curriculum for PGY-1 residents in TY, IM, and EM programs. The curriculum was designed to provide early, structured instruction in foundational ultrasound skills during a formative phase of residency training. Our primary objective was to determine whether this intervention was associated with improved resident comfort immediately after instruction. As an exploratory secondary aim, we included a six-month follow-up survey to describe self-reported later use of POCUS and residents’ perceptions of confidence and clinical benefit over time.

## Materials and methods

Design

This study used a pre- and postintervention observational design evaluating changes in self‑reported comfort across four ultrasound domains using 5‑point Likert items (1 = very uncomfortable, 5 = very comfortable). The four items assessed were: 1) comfort using ultrasound to place a peripheral IV, 2) comfort using ultrasound to aspirate blood or fluid from a vessel or collection, 3) comfort using ultrasound to identify and measure the depth of foreign bodies, and 4) comfort understanding and interpreting echogenicity and echotexture on ultrasound images (see Appendix A).

Participants

Eligible participants included all PGY-1 residents in the TY, EM, and IM residency programs at a single academic community medical center during the study period. Participation was voluntary, and all eligible residents were invited to attend the bootcamp and complete the study surveys. Residents were included in the primary paired pre-/postanalysis only if they completed both the preintervention and postintervention assessments. This project was reviewed by the Saint Agnes Medical Center Institutional Review Board and was determined not to constitute human subjects research.

Sample size considerations

The study sample consisted of all eligible PGY-1 residents from the TY, IM, and EM programs who participated in the curriculum during the study period. Because this project was designed as a pilot educational intervention within a fixed residency cohort, a formal a priori power analysis was not performed. Sample size was therefore determined by participant availability rather than by a prespecified effect size calculation. Accordingly, the findings should be interpreted as exploratory and hypothesis-generating.

Intervention

Participating PGY-1 residents completed a focused introductory POCUS curriculum designed around a microskill-based teaching approach. In this model, complex ultrasound tasks were decomposed into smaller, teachable components that could be practiced individually before being integrated into more complete procedural performance. These microskills included probe grip and hand positioning, probe orientation, image acquisition, depth adjustment, target identification, needle or object visualization, and image optimization.

Prior to participation, residents viewed a brief five-minute prerecorded instructional video covering basic ultrasound machine operation and the targeted skills for each station. Learners then rotated sequentially through four hands-on stations. Each station lasted approximately 15 minutes, for a total of 60 minutes of hands-on practice. Because instruction was delivered in very small groups, no more than two residents participated in a given session. One faculty instructor supervised all four stations as learners rotated sequentially through them, yielding an approximate faculty-to-learner ratio of 1:2 during each session. At each station, the instructor used a graded checklist of station-specific microskills to guide observation and immediate feedback, supplemented by individualized coaching during learner performance (see Appendix B).

Four simulation models were constructed using opaque ballistic gel to approximate human soft tissue and facilitate the development of ultrasound microskills.

Station 1 utilized nasal cannula tubing filled with red dye to simulate vascular structures for ultrasound-guided peripheral IV placement. The learning objectives were probe positioning over a simulated vessel, vessel identification, image centering, maintenance of target visualization, and coordination of needle visualization with probe movement.

Station 2 incorporated paper clips, water balloons, and sponge materials to teach recognition of echogenicity and echotexture. The learning objectives were identification of differences in ultrasound appearance among materials, adjustment of gain and depth to improve image interpretation, and recognition of basic sonographic texture patterns.

Station 3 featured BBs embedded at varying depths to allow learners to practice identification and depth estimation of foreign bodies. The learning objectives were localization of small echogenic targets, depth measurement, probe repositioning to optimize target visualization, and interpretation of shadowing or artifacts.

Station 4 employed a water balloon filled with a water-shaving cream mixture to simulate a soft-tissue abscess. The learning objectives were recognition of a fluid collection, assessment of surrounding tissue appearance, image optimization, and ultrasound-guided aspiration technique.

Residents completed pre- and postintervention surveys following the curriculum, and a six-month follow-up survey was distributed after the intervention.

Statistical analysis

For each Likert-scale item, paired pre- and postintervention responses were analyzed using paired t-tests. Mean differences and corresponding 95% confidence intervals (CI) were calculated to quantify the magnitude and precision of change. Effect sizes were reported as Cohen’s dz for paired samples. Because Likert-scale responses are ordinal and the sample size was modest, Wilcoxon signed-rank tests were additionally performed as a nonparametric sensitivity analysis. A two-sided p value of <0.05 was considered statistically significant. All statistical analyses were conducted using Microsoft Excel (Microsoft Corp., Redmond, WA).

Follow‑up survey (six‑month assessment)

As an exploratory six-month follow-up assessment, all participating PGY-1 residents were invited to complete a voluntary survey six months after the initial curriculum (see Appendix C). The survey assessed self-reported confidence with foundational ultrasound skills, including basic ultrasound image acquisition, identification of needle tip during ultrasound-guided procedures, and adjustment of probe orientation and image settings. It also assessed the frequency of POCUS use in clinical practice, perceived impact on clinical efficiency, confidence using ultrasound in patient care, and perceived ability to integrate ultrasound into clinical decision-making. All follow-up responses were anonymous and aggregated.

## Results

Participant characteristics

Fourteen PGY-1 residents from TY, IM, and EM training programs at Saint Agnes Medical Center in Fresno, CA, completed both the pre- and postintervention assessments and were included in the paired analysis.

Immediate postintervention outcomes

Mean self-reported comfort scores increased across all four ultrasound domains following the instructional intervention (Table 1). For comfort using ultrasound to place a peripheral IV (Q1), the mean score increased from 2.36 ± 1.01 pretraining to 3.29 ± 1.27 posttraining, with a mean difference of 0.929 (95% CI, 0.573-1.284; p < 0.001). For comfort using ultrasound to aspirate blood or fluid from a vessel or collection (Q2), the mean score increased from 2.64 ± 1.08 to 3.36 ± 1.08, with a mean difference of 0.714 (95% CI, 0.238-1.191; p = 0.006). For comfort using ultrasound to identify and measure the depth of foreign bodies (Q3), the mean score increased from 2.14 ± 1.23 to 3.86 ± 0.77, with a mean difference of 1.714 (95% CI, 1.187-2.242; p < 0.001). For comfort understanding and interpreting echogenicity and echotexture on ultrasound images (Q4), the mean score increased from 2.29 ± 0.99 to 4.07 ± 0.92, with a mean difference of 1.800 (95% CI, 1.371-2.229; p < 0.001). Wilcoxon signed-rank testing showed a similar pattern of statistical significance across all four domains, supporting the consistency of the results despite the ordinal nature of the Likert-scale responses. Effect sizes were large for all items (Cohen’s dz range: 0.865-2.324), with the greatest effects observed for foreign body identification and depth estimation (Q3) and echogenicity/echotexture interpretation (Q4); Q4 showed the greatest mean improvement overall.

**Table 1:** Pre-post differences in self-reported comfort across four ultrasound skill domains (n = 14) Pre- and postintervention changes in resident comfort scores following the POCUS microskills curriculum SD: standard deviation; CI: confidence interval; POCUS: point-of-care ultrasound; IV: intravenous

Domain	Pre, mean ± SD	Post, mean ± SD	Mean difference	95% CI	p value
Comfort using ultrasound to place a peripheral IV (Q1)	2.36 ± 1.01	3.29 ± 1.27	0.929	0.573-1.284	<0.001
Comfort using ultrasound to aspirate blood or fluid from a vessel or collection (Q2)	2.64 ± 1.08	3.36 ± 1.08	0.714	0.238-1.191	0.006
Comfort using ultrasound to identify and measure the depth of foreign bodies (Q3)	2.14 ± 1.23	3.86 ± 0.77	1.714	1.187-2.242	<0.001
Comfort understanding and interpreting echogenicity and echotexture on ultrasound images (Q4)	2.29 ± 0.99	4.07 ± 0.92	1.800	1.371-2.229	<0.001

Six-month follow-up outcomes

Seven of the fourteen trained residents completed the follow-up survey (see Appendix C). Among respondents, the majority reported favorable confidence ratings in foundational ultrasound skills, including image acquisition and needle-tip identification. Respondents also reported ongoing use of POCUS in clinical practice, and most endorsed that ultrasound improved their clinical efficiency and confidence in patient care. Several respondents also reported that the microskills curriculum improved their ability to integrate ultrasound into clinical decision-making. Given the limited follow-up sample and the potential for nonresponse bias, these findings should be interpreted as exploratory and descriptive.

## Discussion

This study demonstrates that a structured POCUS curriculum using a microskill-based teaching approach was associated with significant improvements in learner comfort across four ultrasound domains. Rather than asking novice learners to perform fully integrated ultrasound tasks from the outset, this curriculum decomposed complex skills into smaller, teachable components practiced in a stepwise fashion. This approach is consistent with prior work showing that structured and simulation-based ultrasound instruction can improve learner confidence and procedural skill development [3-5].

The curriculum’s emphasis on microskills aligns with established psychomotor frameworks describing the visuomotor and visuospatial demands of ultrasound imaging [1,2]. Breaking down complex ultrasound tasks into smaller, discrete elements is consistent with contemporary instructional models for psychomotor learning and has demonstrated effectiveness in procedural domains requiring fine motor control, sequencing, and deliberate practice [2,6]. In other technical fields, microskill-based teaching has been shown to support skill acquisition, improve performance, and facilitate progression along the learning curve [7]. Our findings suggest that a similar stepwise instructional model may be useful in ultrasound education, where learners must integrate hand positioning, probe orientation, image acquisition, target identification, and image optimization into coordinated performance.

Several limitations should be considered when interpreting these findings. First, this was a small, single-center pilot study with voluntary participation, which introduces the possibility of selection bias. Not all eligible PGY-1 residents participated, and the sample may have disproportionately represented residents with greater baseline interest in ultrasound. As a result, the findings may not be fully generalizable across all residency programs represented in the invited cohort. Second, the primary outcome was self-reported comfort rather than objective competency, image quality, or procedural success. Although improved comfort is educationally relevant, it does not necessarily equate to improved technical performance. This also raises the possibility that self-reported comfort may exceed objective technical proficiency.

An additional limitation is the low response rate for the six-month follow-up survey. Only seven of the 14 residents who completed the initial curriculum responded, representing a 50% loss to follow-up. Because the follow-up survey was voluntary, anonymous, aggregated, and self-reported, these data are subject to substantial nonresponse bias, as residents who perceived the curriculum to be more useful may have been more likely to respond. Moreover, because six-month responses could not be linked to each resident’s earlier survey results, we could not determine whether improvements were maintained at the individual level over time. Accordingly, the six-month findings should be interpreted as exploratory and descriptive rather than as evidence of durable training effects or confirmed translation into clinical practice. Future studies should consider using unique anonymized identifiers to permit paired longitudinal follow-up and better evaluate maintenance or decay of these skills over time.

Despite these limitations, the observed short-term improvement in self-reported comfort suggests that a microskill-based, stepwise instructional approach may be a useful framework for teaching foundational ultrasound skills. Future studies should evaluate this model in larger cohorts with broader participation across training programs, objective competency-based outcomes, and more rigorous longitudinal follow-up. A randomized trial incorporating blinded expert ratings of image acquisition and procedural performance would provide a stronger test of the educational impact of this curriculum.

## Conclusions

Targeted ultrasound instruction delivered through a microskill-based teaching approach was associated with significant and educationally meaningful improvements in PGY-1 resident comfort across four domains. Structured, stepwise practice of discrete psychomotor ultrasound skills may help learners develop the confidence, coordination, and image-acquisition abilities needed for more complex clinical applications.

This microskill-based model represents a pragmatic and scalable framework for foundational ultrasound education. Future studies should evaluate this approach using objective competency-based outcomes, including blinded image quality ratings in a randomized trial.

## Appendices

Appendix A

**Table 2:** Pre- and postintervention ultrasound comfort questionnaire Questionnaire used to assess resident self-reported comfort across four ultrasound domains before and after the microskills curriculum Credit: The questionnaire was created by the authors

Question number	Survey item	Response options
Q1	How comfortable are you using ultrasound to place a peripheral IV?	5-point Likert scale (1 = Very uncomfortable, 5 = Very comfortable)
Q2	How comfortable are you using ultrasound to aspirate blood or fluid from a vessel or collection?	5-point Likert scale (1 = Very uncomfortable, 5 = Very comfortable)
Q3	How comfortable are you using ultrasound to identify and measure the depth of foreign bodies?	5-point Likert scale (1 = Very uncomfortable, 5 = Very comfortable)
Q4	How comfortable are you in understanding and interpreting echogenicity and echotexture on ultrasound images?	5-point Likert scale (1 = Very uncomfortable, 5 = Very comfortable)

Appendix B

**Table 3:** Faculty checklist Faculty checklist used during microskills lab stations IV: intravenous Credit: The questionnaire was created by the authors

Station	Checklist item	Response
-	Resident Name	-
-	Specialty	-
Station 1. IV placement and aspiration	Q1. Vessel identification: were you able to identify and visualize a compressible vessel?	-
Station 1. IV placement and aspiration	Q2. Aspiration skill: once the vessel was identified, were you able to successfully aspirate blood, confirming intravascular placement?	-
Station 1. IV placement and aspiration	Q3. Cannula advancement: after aspiration, were you able to successfully advance the cannula into the vessel and withdraw the needle without dislodging?	-
Station 2. Tissue skills	Q1. What is the echogenicity (hypoechoic, hyperechoic, or anechoic) of the items in each model (A, B, C)?	-
Station 2. Tissue skills	Q2. What is the echotexture (heterogeneous or homogeneous) of the items in each model (D, E)?	-
Station 3. Depth and foreign bodies	Q1. How many foreign bodies did you find?	-
Station 3. Depth and foreign bodies	Q2. Measure and record the depth of any foreign body	-
Station 4. Abscess aspiration	Q1. Were you able to successfully aspirate?	-

Appendix C

**Table 4:** Six-month follow-up POCUS questionnaire The questionnaire used to assess resident comfort, confidence, and clinical utilization of point-of-care ultrasound following the microskills curriculum POCUS: point-of-care ultrasound Credit: The questionnaire was created by the authors

Question number	Question	Response options
1	How comfortable are you performing basic ultrasound image acquisition?	1-5 Likert scale (Very uncomfortable → Very comfortable)
2	How confident are you identifying needle tip during ultrasound-guided procedures?	1-5 Likert scale
3	How comfortable are you adjusting probe orientation and optimizing image settings?	1-5 Likert scale
4	How frequently do you use point-of-care ultrasound during clinical shifts?	Never/Rarely/Sometimes/Often/Always
5	Do you feel that POCUS improves your clinical efficiency?	Yes/No
6	Do you feel more confident using ultrasound in patient care after completing the curriculum?	Yes/No
7	Six months after the training, how frequently are you using POCUS in clinical practice?	Never/Rarely/Sometimes/Often/Always
8	Do you feel that the microskills curriculum improved your ability to integrate ultrasound into clinical decision-making?	Yes/No
